# Flexible intramedullary nailing in paediatric femoral fractures. A report of 73 cases

**DOI:** 10.1186/1749-799X-6-64

**Published:** 2011-12-22

**Authors:** Ramprakash Lohiya, Vikas Bachhal, Usman Khan, Deepak Kumar, Vishwapriya Vijayvargiya, Sohan S Sankhala, Rakesh Bhargava, Nipun Jindal

**Affiliations:** 1 Department of Orthopaedics, All India Institute of Medical Sciences, New Delhi 110029, India; 2 Registrar, Department of Orthopaedics, Postgraduate Institute of Medical Education and Research, Chandigarh 160012, India; 3 Sawai Man Singh Medical College and Hospital, Sawairam Singh Road, Jaipur 302004, Rajasthan, India

## Abstract

**Background:**

Flexible intramedullary nailing has emerged as an accepted procedure for paediatric femoral fractures. Present indications include all patients with femoral shaft fractures and open physis. Despite its excellent reported results, orthopaedic surgeons remain divided in opinion regarding its usefulness and the best material used for nails. We thus undertook a retrospective study of paediatric femoral fractures treated with titanium or stainless steel flexible nails at our institute with a minimum of 5 years follow up.

**Material and methods:**

We included 73 femoral shaft fractures in 69 patients treated with retrograde flexible intramedullary nailing with a minimum follow up of 5 years. Final limb length discrepancy and any angular or rotational deformities were determined.

**Results:**

Mean age at final follow up was 15.5 years (10-21 years). Mean follow up was 7.16 years (5.0-8.6 years). Titanium and stainless steel nails were used in 43 and 30 cases respectively. There were 51 midshaft, 17 proximal, and 5 distal fractures.

All fractures united at an average of 11 weeks but asymptomatic malalignment and LLD were seen in 19% and 58% fractures respectively. LLD ranged from -3 cm to 1.5 cm. Other complications included superficial infection(2), proximal migration of nail(3), irritation at nail insertion site(5) and penetration of femoral neck with nail tip(1). There were 59 excellent, 10 satisfactory and 4 poor results.

**Conclusion:**

Flexible intramedullary nailing is reliable and safe for treating paediatric femoral shaft fractures. It is relatively free of serious complications despite asymptomatic malalignment and LLD in significant percentage of fractures.

## Introduction

After acute infections, trauma is a leading cause of morbidity and mortality in children [1,2]. Although accounting for less than 2% of all orthopaedic injuries in children [3], femoral fractures have a significant impact not only on the patient and their family network, but also on regional trauma resources [4,5]. These fractures have been managed with wide variety of methods in past. Historically treatment with closed means in plaster spica cast, either immediately or after a period of traction, has yielded acceptable results for these fractures [6-8] but this treatment produces undue physical and psychological stress for patient and family [9-11]. Furthermore, in certain complex fractures and sometimes in subtrochantric fractures, with tendency for marked flexion of proximal fragment, closed reduction and its maintenance if often unsuccessful. Last few decades has seen increasing trend towards operative management of femoral shaft fractures in paediatric patients but opinion regarding optimal method of fixation of these fractures remains divided [12]. External fixation, although producing acceptable results, is fraught with many complications as is plate osteosynthesis and rigid intramedullar nailing which may also require a second major surgery for removal of implant [13-21]. Flexible intramedullary nailing introduced for femoral fractures by Nancy group in 1982 [22], has become popular with many orthopaedic surgeons and remains the treatment of choice for these fractures at our institute due to its favourable results and lack of serious complications.

We undertook a long term retrospective study of paediatric femoral fractures treated with flexible intramedullary nailing at our institute.

## Materials and methods

On retrospective search of hospital records, we found 81 patients of femoral shaft fractures treated with flexible intramedullary nailing at our institute with a minimum follow up period of 5 years. All patients with open fractures, pathological fractures, metabolic bone disease or neuromuscular disorders were excluded from search. Of these 81 patients, 69 patients with 73 femoral shaft fractures were available for follow up. Indication for surgery in all cases was displaced femoral shaft fracture with open femoral physis. A written informed consent was obtained from each patient or their family for inclusion in this study. There were 53 males (57 fractures) and 16 females (16 fractures) in this series with an average age of 8.3 (range 4-15) years at the time of injury (Table 1). Fracture locations were 51 midshaft, 17 proximal, and 5 distal fractures. Fracture patterns included transverse (49), oblique (21), and communited (3) fractures. Fractures were classified according to system of Winquist [23] as Grade I (45), Grade II (14), Grade III (11) and Grade IV (3) (Table 2). All cases were operated within first 6 (mean 2.3) days of injury.

**Table 1:** Demographics

Patients treated	81
**Patients followed**	69

**Fractures followed**	73

**Male:Female**	53(57 fractures):16(16 fractures)

**Mean age**	8.3 years(4-15)

**Nail used**	

*TENS*	43

*Enders*	30

**Table 2:** Fracture characteristics

Location	
*Proximal*	17

*Midshaft*	51

*Distal*	5

**Pattern**	

*Transverse*	49

*Oblique*	21

*Communited*	3

**Winquist grading**	

*I*	45

*II*	14

*III*	11

*IV*	3

All surgeries were performed on fracture table under radiographic control. Two prebent flexible nails were inserted across the fracture in a retrograde fashion. Although, fracture reduction was attempted with closed means in all cases but open reduction had to be done in 12 cases. Both nails were inserted about 2 cm proximal to distal femoral physis from medial and lateral sides. Medial nail was directed till it was within 2 cm of proximal femoral capital physis whereas lateral nail was inserted till it was about 1 cm from greater trochantric physis. Nail diameter was predetermined as being able to fill 40% of medullary canal at the level of isthimus but in practice intraoperative decision regarding nail diameter was taken by operating surgeon. Titanium elastic nails were used in 43 fractures while stainless steel nails in 30 fractures. All titanium nails were bent at insertion site and cut close to bone leaving 1.5-2 cm of nail protruding for later easy removal. Stainless steel nails (Ender's nails) had an eye at distal end which was used for extraction and thus allowed us to advance it relatively flush with bone. After completion of procedure, rotational stability was assessed in all cases by rotating distal fragment under radiographic control. Average operative time for this procedure was 37 (range 25-110) minutes. Under usual circumstances, most patients were discharged within 2-3 days postoperatively after inspection of surgical site. Average hospital stay for patients was 5.1 (3-9) days.

Although no postoperative immobilisation was routinely used, however 3 cases with Winquist Grade IV fractures were put in hip spica cast for initial 4 weeks for achieving better stability at fracture site. The decision regarding use of postoperative immobilisation was based entirely on discretion of the operating surgeon who was thought to be the best judge of stability achieved at fracture site after surgery. We routinely checked for stability of fractures by moving and stressing the fracture under image intensifier and fractures thought to be unstable were immobilised for 4-8 weeks postoperatively. Five additional cases with grade III communition were immobilised with spica cast or knee immobiliser for 4 weeks. Three of these 5 fractures were distal and 2 involved midshaft region. The purpose of post-operative immobilization was to provide extra stability at fracture site in cases where flexible nailing was unable to achieve adequate stability as demonstrated by rotating the distal segment under radiographic control. This method tested for rotational stability. Apart from these cases, 3 cases with grade IV comminution were deemed to be axial unstable as well and thus immobilized. Postoperative rehabilitation included hip and knee mobilisation on first postoperative day followed by partial weight bearing after significant pain and inflammation has resolved after 3-4 days. Weight bearing was delayed in cases with significant communition (Winquist Grade II and above) till signs of callus formation were evident on follow up radiographs. Weight bearing was again delayed for all four bilateral cases regardless of the level of commuinition at fracture site. Progression of union at fracture site was monitored on serial radiographs, usually taken at intervals of 4 weeks, and full weight bearing was allowed once radiographic union was achieved.

**Figure 1:**
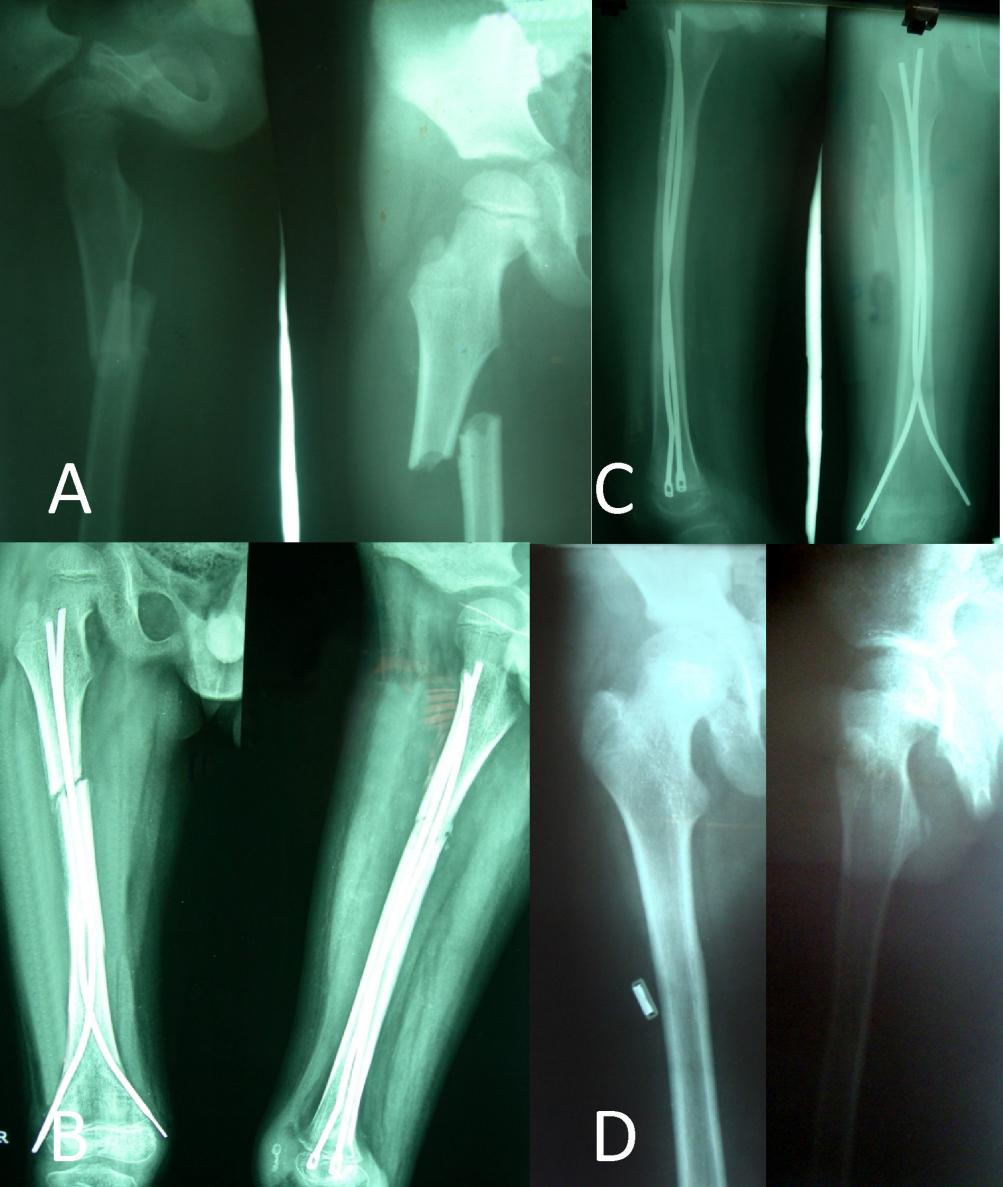
**Femoral shaft fracture of right side in a 6 year old child treated with Ender's nail.** Radiographs revealed displaced femoral shaft fracture (A) of right side. Excellent fracture reduction (B) was achieved which was maintained till fracture union (C) and final follow up radiographs at 6 years postoperatively (D) demonstrated neutral alignment in both anteroposterior and lateral views

**Figure 2:**
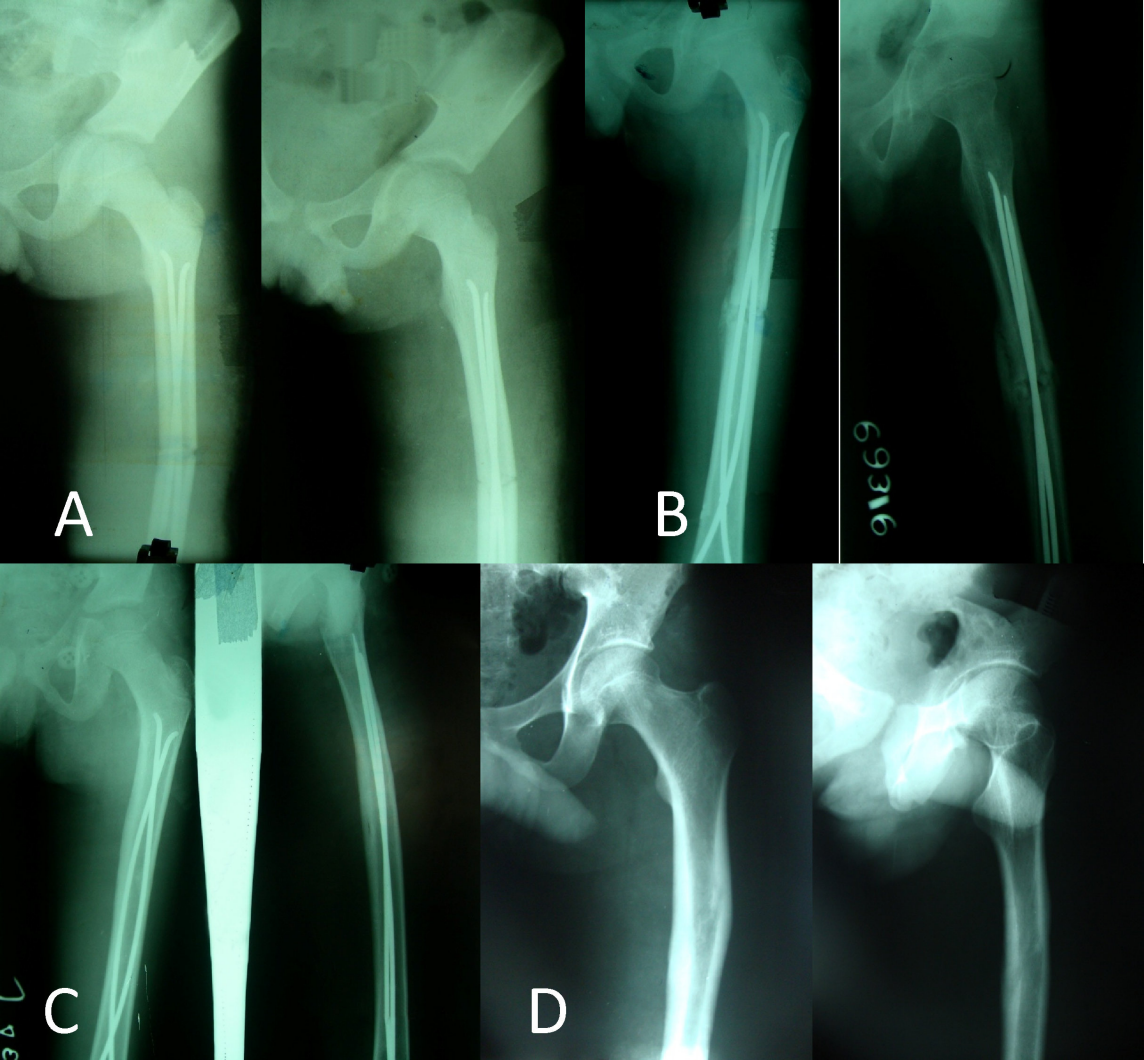
**Radiographs of a 9 year old child with left sided femoral shaft fracture managed by titanium flexible nailing. **(A) shows significant malalignment in both coronal (15° varus) and saggital plane (10° anterior apex). Fracture alignment improved slightly during follow up (B, C) and at 7 years significant malalignment still remained in coronal plane (15°) but not in saggital plane.

Postoperative radiographs were assessed for nail prominence (measured from nail bone interface to nail tip), and both postoperative and final follow up radiographs were assessed for coronal or saggital malalignment and any obvious implant related or unrelated complication (Figure 1 and 2). Rotational malalignment and limb length discrepancy were assessed clinically at latest follow up (bilateral fractures were excluded from this assessment for obvious reason of lack of normal comparison). Significant malalignment was defined as > 10° in coronal plane and > 15° in saggital plane. We routinely removed the nails after achieving solid union although 3 patients failed to show up for routine follow up visits in time for nail removal resulting in proximal migration of nail insertion site with continued growth from distal femoral physis. All fractures were rated according to the system described by Flynn as excellent, satisfactory or poor [24].

## Results

Mean age of patients after an average follow up of 7.16 (range 5.0-8.6) years was 15.5 (range 10-21) years. During serial radiographic monitoring for fracture union, early callus was seen on an average of 3.8 (range 2-6) weeks after surgery and full radiographic union was achieved at 11 (range 6-18) weeks without further intervention. Post-operative immobilisation was used in 8 fractures. Early weight bearing was allowed in 45 cases with Winquist Grade I fracture while in remaining fractures it was delayed variably depending on progression of union at fracture site. Mean time for achieving unassisted full weight bearing in these 45 cases was 10.5 weeks as compared to 15 weeks for remaining cases. Nail removal was done in 70 fractures at an average of 11 (5-16) months postoperatively. All patients regained full range of motion of knee and hip after removal of nails.

Average nail prominence for titanium nails on medial and lateral sides was 17.5 (8-24) mm and 19.2 (12-27) mm respectively whereas same values for ender's nails were 9 (4-15) mm and 12.6 (4-18) mm respectively. There was a significant difference in nail prominence between ender's nail and titanium nail (p < 0.01).

Angulation measured at final follow up in both coronal and saggital planes revealed significant malalignment in 3 cases however minor malalignment was observed in 29 cases. In contrast rotational malalignment was detected in 11 patients (17%) although this was measured clinically by comparing hip rotations to opposite normal limb in cases with unilateral fractures (n = 65). There was a significant relation between angular malalignment with severity of communition as 11 out of 14 patients (71.5%) with winquist grade III or IV fractures had malalignment as compared to 21 out of 59 in grade I or II (chi square, p < 0.01). We did not find any statistically significant difference in malalignment along any axis between types of nails (p = 0.30). We further did not find any significant relation between location of fracture and degree of malalignment (p = 0.361), however the method of treatment differed amongst these groups as postoperative spica was used more frequently for subtrochantric and distal femoral fractures (p < 0.01).

Limb length discrepancy, measured clinically in unilateral cases (n = 65), ranged from -3 to 1.5 cm and revealed unequal limb lengths in 38 patients (58%) with lengthening in 32 patients and shortening in 6 patients. However, 25 of these 38 patients had LLD of less than 1 cm and there were no functional problems reported due to this inequality in length. Of the remaining 13 patients 5 had shortening and 8 had lengthening of fractured limb. All cases of shortening occurred in Winquist grade III or IV. There was no statistically significant difference in limb length discrepancy between the nail types (p = 0.21).

**Figure 3:**
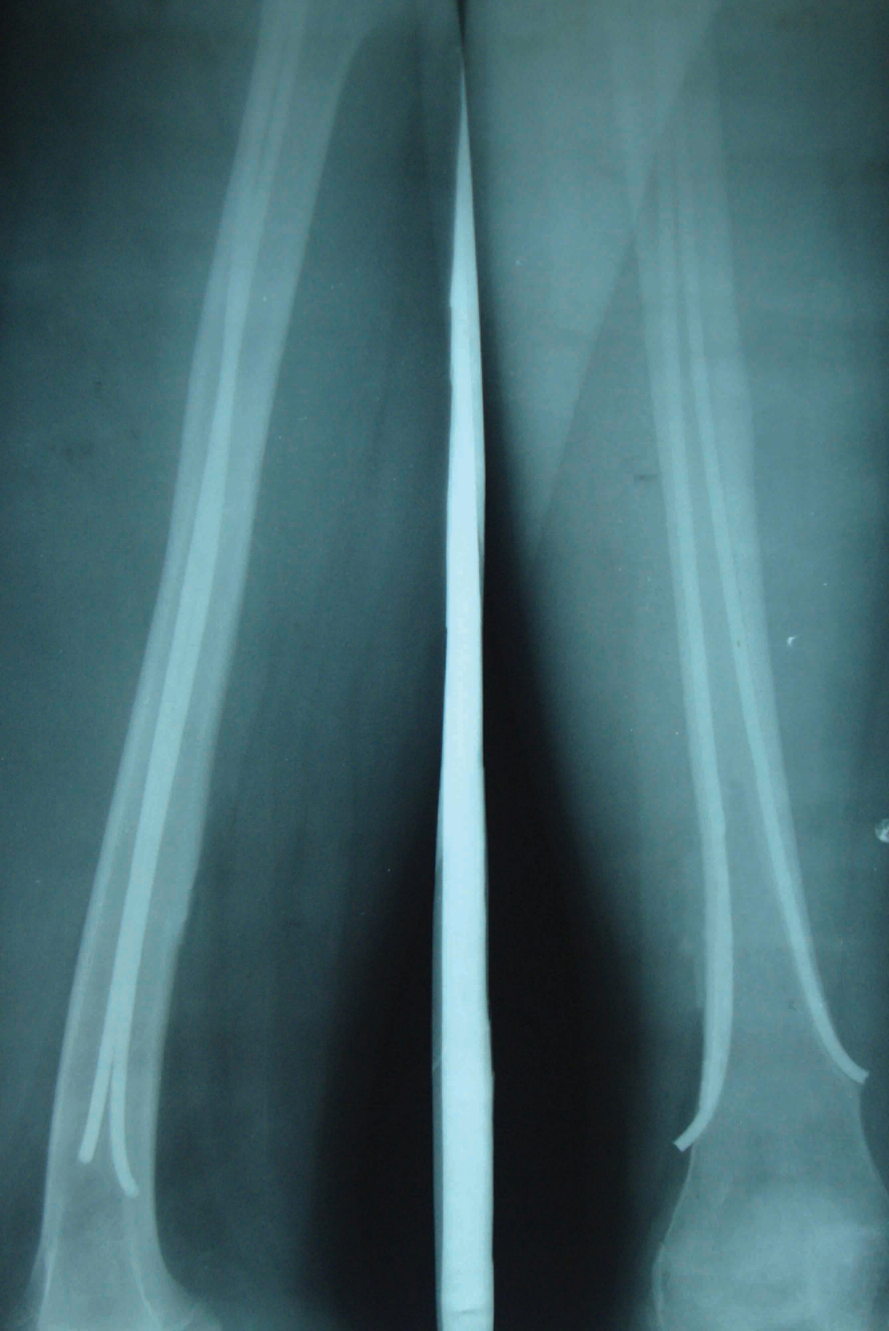
**Proximal migration of nail insertion site due to growth from distal femoral physis**. Patient remained asymptomatic.

**Figure 4:**
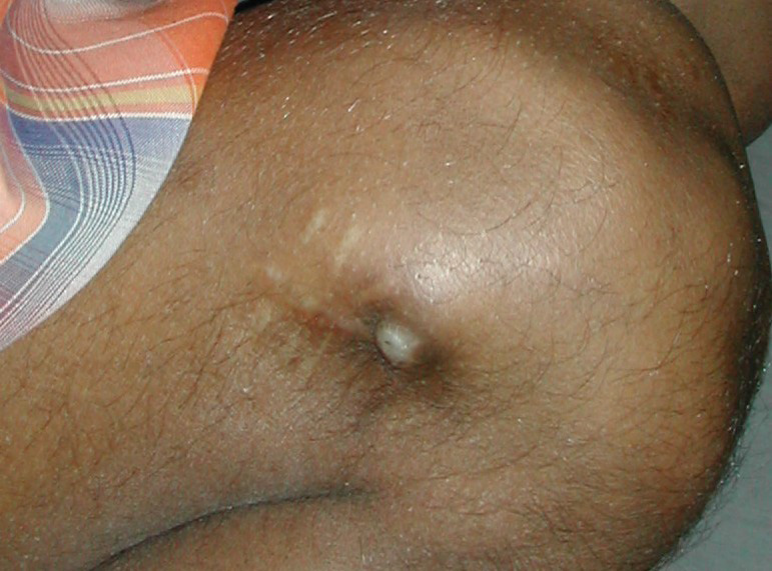
**Superficial skin breakdown at lateral nail insertion site of titanium flexible nail**.

**Figure 5:**
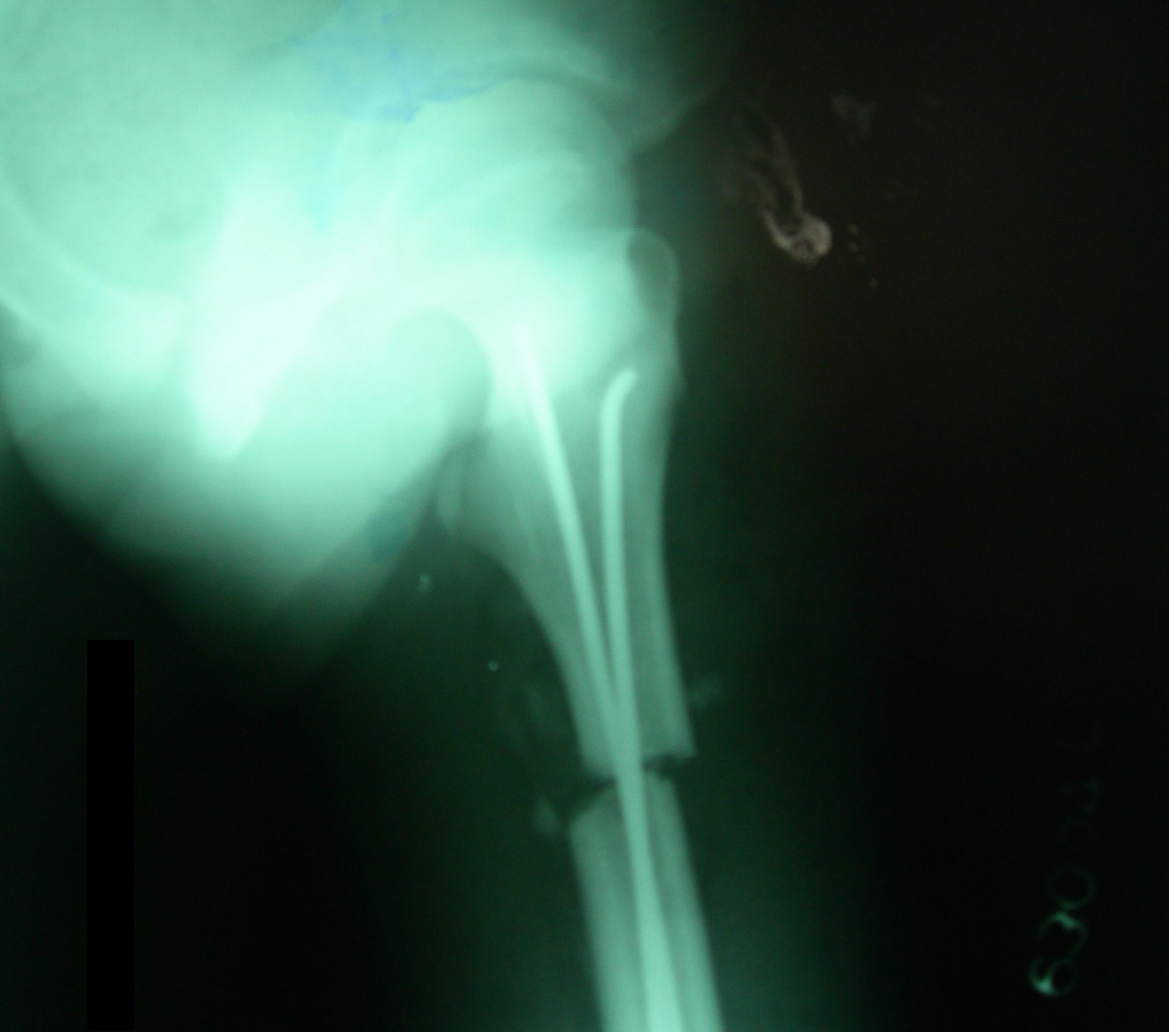
Lateral radiographs of a patient showing good nail position in proximal femur on immediate postoperative radiographs

**Figure 6:**
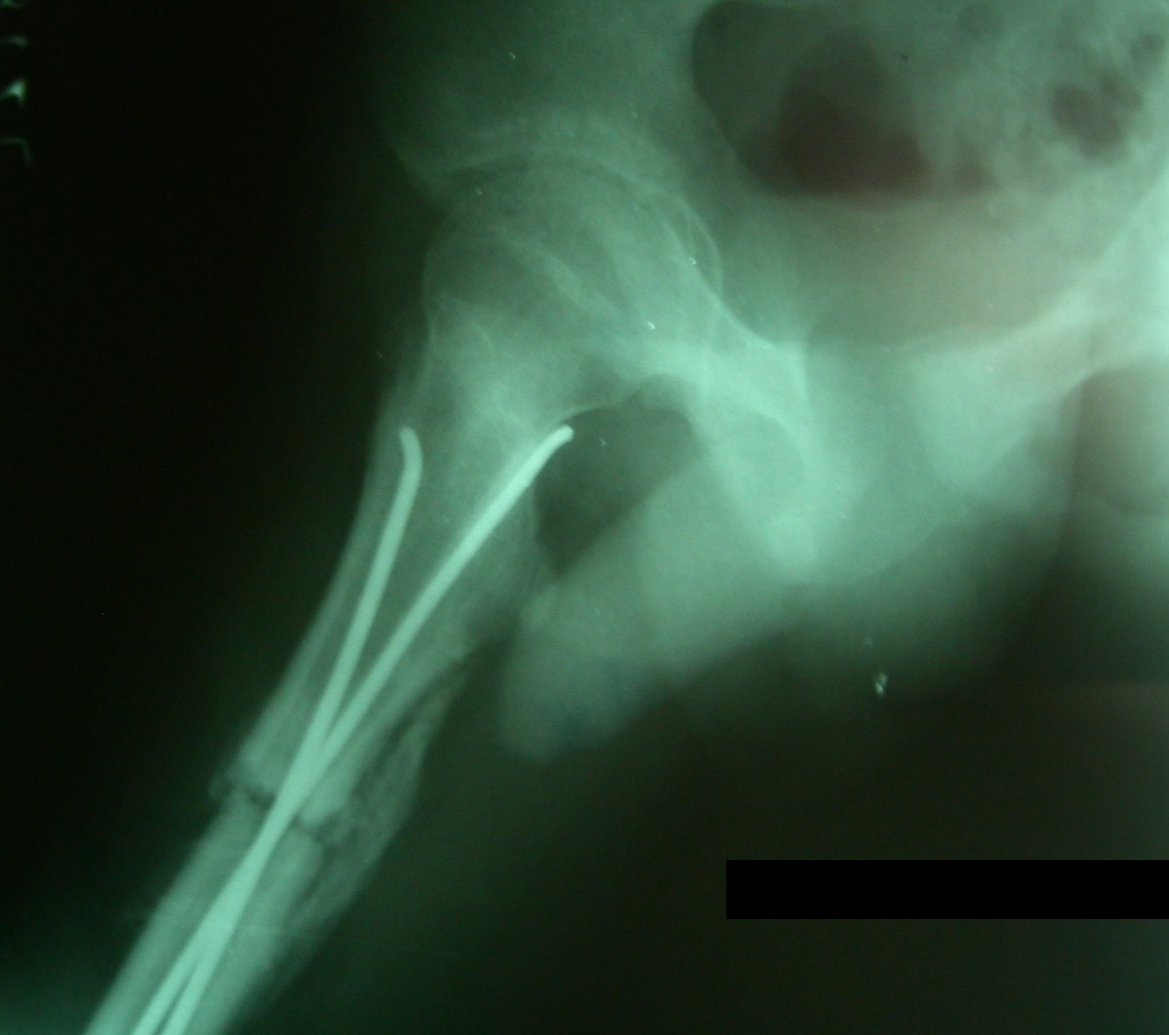
**Lateral radiographs of patient in figure 5 at 5th postoperative week revealed penetration of posteromedial by medial nail, nail was retained till full union**.

Other complications (Table 3) included two cases of superficial infection treated with a prolonged course of antibiotics and 3 cases with proximal migration of nail insertion site following continued distal femoral growth due to failure to remove nails in time although no long term complication occurred in these cases (Figure 3). There was no case of physeal damage. One of the most frequent complaints of patients was irritation at nail insertion site due to prominence of nail leading to bursitis in 5 patients (Figure 4) which resolved after removal of nails but these complaints were significantly more frequent for titanium nails as compared to ender's nail (p < 0.01). In one case, tip of medial nail was found to have penetrated the cortex posteriorly but this was discovered at 5^th ^postoperative week when fracture already demonstrated bridging callus (Figure 5 and 6) and this nail was retained till full union and later removed at 22 postoperative weeks. Proper imaging under C arm with images in different degree of rotation can avoid this complication as even perfectly aligned good quality postoperative radiographs might not be able to reveal all such cases. There was no case of long term knee or hip stiffness although 55.8% cases had some degree of restriction of knee movements before removal of nails which was done at an average of 11 months postoperatively. According to the criterion of Flynn et al there were 59 excellent, 10 satisfactory and 4 poor results. There was no difference in results with type of nail used in this series (p = 0.12) (Table 4).

**Table 3:** Complications

Malunion (coronal/saggital)	3
**Limb length disrepency**	13

**Superficial infection**	2

**Proximal migration**	3

**Bursitis at nail insertion site**	5

**Perforation of cortex of femoral neck**	1

**Table 4:** Results according to nail type

	Excellent	Satisfactory	Poor	Total
**TENS**	37	3	3	43

**Ender's Nail**	22	7	1	30

**Total**	59	10	4	73

## Discussion

Paediatric femoral shaft fractures had been traditionally treated with non operative methods with traction and spica cast application [6-8], however over the past two decades operative treatment has been increasingly tried in order to avoid prolonged immobilisation and other complications of earlier methods. Most popular of these operative treatments have been internal fixation with plate [25-27], rigid fixation with intramedullary nail [28], external fixation [29,30] and more recently flexible intramedullary nailing [24]. Each of these methods has its advantages and disadvantages. External fixation has been associated with refracture and pin-tract infection [31], solid intramedullary nailing with avascular necrosis of the femoral head [18,31,32], thinning of the femoral neck [21] and growth arrest of the greater trochanter with secondary coxa valga [17,21]. In addition, plating of the femur demands extensive soft-tissue dissection and has been related with hardware failure, infection and greater blood loss [31]. Flexible intramedullary nailing, by allowing micromotion at fracture site, promotes bone healing without violating open physis and, being a closed procedure, has a low risk of infection.

Flexible nails had been used for fixation of peritrochanteric fractures with some success [33,34] but its application for paediatric shaft fractures was popularised by nancy team [22]. Since then several authors have reported on the results and complications of this technique. The earlier indication of this technique for femoral shaft fractures was in patients of 6-16 years age group but several authors have reported excellent results in preschool children too [35,36]. Perhaps operative indications for femoral shaft fractures can be expanded to include children of all ages with femoral shaft fractures and open physis.

Flexible nailing for paediatric femoral shaft fractures has yielded predictably excellent union across the literature. Ligier et al reported union in all 123 cases treated with this technique [22]. Flynn et al [24] (n = 58) and Narayanan et al [12] (n = 79) also did not report any union difficulties. Luhmann et al [37] observed one hypertrophic non union in 43 treated femoral shaft fractures. Postoperative immobilisation has been variably used after internal fixation with flexible nails. Ligier et al [22] did not use any postoperative immobilisation in contrast to selective use of spica cast or knee immobilisers by Flynn et al [24] (41/58), Luhmann et al [37] (17/38), Moroz et al [38] (201/234). We used postoperative immobilisation in 8 patients only since adequate fracture stability was achieved in all other cases. Degree of communition was a clear predictor of use of postoperative immobilisation in this series as all such cases were either winquist grade III or IV.

Although most authors have recommended routine nail removal after union however few have recently questioned this practice. Morshed et al in a retrospective study involving 25 fractures treated with TENS reported survivorship free of revision due to pain of 72% at 5 years follow up [39]. Timing of nail removal after fracture union has not been uniform amongst previously published series and there are no clear guidelines in literature. Although early removal has led to occasional complication [24], however many authors have reported satisfactory outcome even after removal of nails as early as beginning of third postoperative month [22]. Overall, most authors have typically recommended nail removal after fracture healing at 6 months to 1 year following surgery [39]. We didn't encounter any difficulty in nail removal even after 1 year and routinely advised our patients for this procedure. Three cases refused for nail removal and demonstrated proximal migration of nail insertion site due to continued growth from distal femoral physis although it did not result in any long term complication.

It has been recommended that diameter of each nail should measure 40% of narrowest diameter of the medullary canal [24] and both nails should be of same diameter [12]. This recommendation was followed in all our cases although it was not always possible to follow 40% rule. Use of stainless steel nails has not been recommended in past because of fear of malunion owing to more stiffness of steel compared to titanium. This view has been refuted by Wall et al [40] who reported higher malunion rates for titanium nails as compared to similarly designed stainless steel nails. In our study we did not find any statistically significant difference in rates of minor or major malunions between nail types whereas steel nails were considerably cheaper than their titanium counterparts. However, there was a significant difference in malunion rates with degree of communition at fracture site. Narayanan et al [36] and Sink et al [41] reported similar effect of communition on malunion rates.

A frequent complication in this series was skin irritation and pain at nail insertion site leading to limited range of knee movement which resolved completely after nail removal. Similarly high incidence of this minor complication has been observed in previous reports [12,37]. We observed a significant difference in rate of this complication between TENS and ender's nail which was related to tendency of leaving nail flush with femoral cortices in latter. This was perhaps the result of less apprehension of difficulty in nail retrieval in ender's nail, which has an eye (hole) at its end for extraction, as compared to TENS and was not a result of material properties of nails. Narayanan et al [12] has recommended cutting the ends short and advancing the nails with a hollow tamp until the ends lay adjacent to the supracondylar flare of the distal femoral metaphysic but this is not universally practiced technique [24,37]. Moreover, in their series, Narayanan et al [12] did not remove majority of nails which were cut flush with cortex and their opinion regarding ease of nail removal with current instrumentation cannot be validated. Recent introduction of end caps for nails may provide a solution for this problem but our experience with this is not sufficient to make valid observation [42].

Limb length discrepancy was a frequent but clinically insignificant complication as most fractured limbs were within 1 cm in length of the contralateral normal limb. However, shortening of > 1 cm was observed in 5 patients having grade III or IV communition. Although lesser degree of limb length discrepancy is fairly common, however most published articles have reported infrequent occurrence of clinically significant discrepancy [12,24].

Vrsansky et al [43] reported universally good results in 141 fractures without a single complication whereas Flynn et al [24] had only one poor result in 58 fractures. Several other authors have reported variable rates of complications. Sink et al [41] reported 62% complication rate necessitating unplanned surgery before fracture union in third (21%) of these cases. The complications in this series were related to length unstable fractures which were either comminuted or long oblique. In long oblique fractures the length of the obliquity was twice as long as the diameter of the femur at the level of the fracture. Comminuted fractures had more than one continuous fracture and a butterfly fragment. Luhmann et al [37] reported an overall complication rate of 49% (21/43) but only 2 major postoperative complications with rest being minor complications. Ho et al [44] reported a total complication rate of 17%. We had fair share of complications with overall 32 patients reporting significant complication amounting to 44% complication rate. However despite this high rate of complications, there were only 4 poor results in this series with remaining patients having only minor complications.

We conclude that flexible nailing for fracture shaft femur in paediatric age group yields excellent or satisfactory results in majority of patients with reasonable complication rates. Furthermore, stainless steel nails produce results similar to titanium nails at considerably less price.

### Consent statement

Written informed consent was obtained from each patient for publication of this report and accompanying images. Copies of written consents are available for review by the Editor-in-Chief of this journal.

## Competing interests

The authors declare that they have no competing interests.

## Authors' contributions

RL and VB reviewed the literature and wrote the paper. UK, SSS and RB were main operating surgeons in the whole series and critically reviewed the paper. RL, VB, VPV and DK maintained all the records of the patients and followed them. All the authors read and approved the final manuscript.
